# Effectiveness of Acupuncture for Treating Sciatica: A Systematic Review and Meta-Analysis

**DOI:** 10.1155/2015/425108

**Published:** 2015-10-21

**Authors:** Zongshi Qin, Xiaoxu Liu, Jiani Wu, Yanbing Zhai, Zhishun Liu

**Affiliations:** ^1^Department of Acupuncture, Guang'anmen Hospital, China Academy of Chinese Medical Sciences, Beijing 100053, China; ^2^Beijing University of Chinese Medicine, Beijing 100029, China

## Abstract

This is a systematic review and meta-analysis, which aimed to assess the current evidence on the effects and safety of acupuncture for treating sciatica. In this review, a total of 11 randomized controlled trials were included. As a result, we found that the use of acupuncture may be more effective than drugs and may enhance the effect of drugs for patients with sciatica, but because of the insufficient number of relevant and rigorous studies, the evidence is limited. Future trials using rigorous methodology, appropriate comparisons, and clinically relevant outcomes should be conducted.

## 1. Introduction

Sciatica is a syndrome involving nerve root impingement or inflammation that has progressed sufficiently to cause neurological symptoms in the areas that are supplied by the affected nerve roots [1]. The most important symptoms include unilateral leg pain radiating to the foot or toes that is greater than low back pain and often associated with paresthesia, numbness, and weakness of the leg; it may involve increased pain on straight leg raising and neurological symptoms limited to one nerve root. Sciatica may be sudden in onset and may subsequently persist for days or weeks [2, 3].

Frymoyer reported that the prevalence of sciatica varies widely from 13% to 40% [4, 5]. According to the research of Konstantinou, most patients suffered sciatica in the fourth and fifth decades of their life [6]. The treatment for sciatica is primarily aimed at pain control by means of either conservative treatment or surgical techniques. According to the prior systematic review, nonopioid medication, epidural injections, and disc surgery are effective for pain reduction [7]; however, relevant side effects to epidural injections have been reported [8–10], and the effect of NSAIDs on sciatica is still uncertain, even though it is a common treatment to manage pain. Many patients report little relief [7], and the surgical procedures are invasive and expensive and may even cause neurological complications that may not be acceptable for all patients [11].

Acupuncture is a tried and tested system of traditional Chinese medicine, which has been used in China and other Eastern cultures for thousands of years. While acupuncture has been proposed for persistent sciatica, its efficacy has not been shown [7, 12]. To date, there has been substantial research into the anaesthetic and anti-inflammatory actions of acupuncture [13–15], and several randomized controlled trials (RCTs) have suggested that acupuncture can relieve the symptoms of sciatica [16, 17]. Despite these studies, an acupuncture-related systematic review has still fallen short of projected expectations.

This systematic review aimed to assess the current evidence on the effects and safety of acupuncture for sciatica.

## 2. Methods and Analysis

We conducted this systematic review according to a published protocol [18] and our review is reported in accordance with the Preferred Reporting Items for Systematic Reviews and Meta-Analyses (PRISMA) statement [19].

### 2.1. Study Selection

#### 2.1.1. Types of Studies

All randomized controlled trials (RCTs) in English, Chinese, and Japanese on acupuncture treatment for sciatica were included for this review. Non-RCTs, quasi-RCTs, and randomized controlled trial protocol were excluded.

#### 2.1.2. Types of Participants

Patients with sciatica were included, including those diagnosed with sciatica synonyms, such as radiculopathy, nerve root compromise, nerve root compression, nerve root pain, and pain radiating below the knee, with no restriction on gender and age. We excluded trials if they included lower back pain without sciatica.

#### 2.1.3. Types of Interventions

Any type of invasive acupuncture were included, such as acupuncture, electroacupuncture, elongated needle acupuncture, auricular acupuncture, abdominal acupuncture, and warm acupuncture. Control interventions may include no treatment, sham acupuncture/placebo (e.g., acupuncture same acupuncture point without needle insertion or acupuncture the point close to it but it is not an acupuncture point), and Western medicine. As this review aims to assess the effectiveness and safety of acupuncture for treating sciatica, we excluded trials comparing two different types of acupuncture. Furthermore, the effectiveness of Chinese medicine is hard to assess, so we excluded trials comparing acupuncture with Chinese medicine.

#### 2.1.4. Types of Outcome Assessments

The primary outcome of interest was pain intensity. Any validated measurement scales were included (e.g., Visual Analogue Scale (VAS), Numeric Rating Scale (NRS), and Short-Form McGill Pain Questionnaire (SF-MPQ)). Secondary outcomes were (1) global assessment (the proportion of patients improved or cured); (2) quality of life, for example, as assessed using the Medical Outcomes Study 36-Item Short Form health survey (SF-36); (3) physical examinations; (4) patient satisfaction; and (5) adverse effects.

#### 2.1.5. Data Sources

A search strategy was used and conducted according to the Cochrane handbook guidelines [31]. The following nine databases were searched from their inception to May 2015: MEDLINE, EMBASE, CENTRAL, CBM, CMCC, VIP database, Wan-Fang Database, CNKI, and CiNii. The search strategy was based on the guidance of the Cochrane handbook.

The strategy for searching the PUBMED database is shown in Table 1. This search strategy was also applied to the other electronic databases.

#### 2.1.6. Data Extraction

Two authors (Zongshi Qin and Xiaoxu Liu) extracted the data independently. Before beginning extraction, a small scope trial with one database was conducted to confirm that there were no differences between the two authors. After a common understanding was reached, standard extraction forms were used to collect data from included trials. Any disagreements were discussed and judged by an arbiter (Zhishun Liu).

#### 2.1.7. Data Management

Two authors (Zongshi Qin and Xiaoxu Liu) used Endnote X7 (Thomson Reuters, New York, NY, USA) software to manage the trials that have been searched and remove duplicates. Data extracted were put into Revman V.5.3.3 software for analysis.

#### 2.1.8. Risk of Bias in Individual

The Cochrane Collaboration tool for assessing the risk of bias was used to facilitate the assessment of the risk of bias for trials included [32]. Two authors (Jiani Wu and Yanbing Zhai) independently evaluated methodological quality, which covers seven aspects: random sequence generation, allocation concealment, blinding of participants and personnel, blinding of outcome assessment, incomplete outcome data, selective reporting, and other bias. Any disagreements were discussed and resolved by a third author (Zhishun Liu).

#### 2.1.9. Measures of Treatment Effect

Dichotomous data were analysed using risk ratio (RR) and 95% confidence interval (CI). Continuous outcomes were analysed using mean differences (MD) with 95% CI or standardized mean differences (SMD) with 95% CI if different measurement scales are used.

#### 2.1.10. Dealing with Missing Data

The listed corresponding author was contacted in an attempt to obtain any missing information from their trial. We excluded 1 trial after 3 unsuccessful attempts to contact the authors to obtain missing data from the data synthesis [33].

#### 2.1.11. Assessment of Heterogeneity

We used Higgins *I*² statistic to test clinical heterogeneity. Variability factors included in the trials were taken into consideration (e.g., type of intervention and duration of intervention). If *I*²≥50% or *P* < 0.1, there is substantial heterogeneity among the trials, and the design of trials and characteristics in the included trials were analysed.

#### 2.1.12. Assessment of Reporting Biases

A funnel plot was used to assess the reporting biases when 10 or more trials were included in a meta-analysis. However, the number of studies included in our analysis may have been too small to test for funnel plot asymmetry [34].

#### 2.1.13. Confidence in Cumulative Estimate

Details of acupuncture and control interventions were extracted on the basis of the revised Standard for Reporting Interventions in Clinical Trials of Acupuncture (STRICTA) [35], a checklist intended for use in conjunction with CONSORT that can estimate randomized controlled trials of acupuncture, including acupuncture rationale, needling details, treatment regimen, cointervention, control interventions, and treatment background. The acupuncture interventions in the included studies based on the STRICTA recommendations are presented in Table 2.

#### 2.1.14. Data Synthesis

We used Revman V.5.3.3 software to perform meta-analysis of the trials included. Dichotomous data were determined by using RR with 95% CI, and continuous outcomes were analysed using WMD with 95% CI or SMD with 95% CI if different measurement scales are used. When statistical heterogeneity was observed, the random effects model was used; otherwise the fixed effect model was used to combine the data. When quantitative synthesis was not appropriate, we provided systematic narrative synthesis to describe the characteristics and findings of the included trials.

#### 2.1.15. Subgroup Analysis and Sensitivity Analysis

We planned to conduct subgroup and sensitivity analyses in the published protocol as follows: we hypothesized a greater reduction in pain intensity and improvement in global assessment with acupuncture than with sham acupuncture; we also predicted that different types of sciatica or risks of bias in different trials would lead to moderate statistical heterogeneity.

## 3. Results

### 3.1. Selection of Studies

Our search strategy yielded a total of 1489 records. After 435 duplicate records were excluded, 1054 unique records were screened for eligibility. A total 1005 records were excluded based on review of the title and abstract. The remaining 49 records were deemed potentially relevant. After the full-text articles were reviewed, 7 studies were excluded because they were not true RCTs, 24 studies were excluded because they included inappropriate interventions, and 7 studies were excluded due to inappropriate design. One study was published in French and the full-text was unavailable; thus, we were unable to extract the data, and the study was therefore excluded from review [36]. In total, 11 studies met the criteria predesigned in our protocol and were therefore included in our review for systematic and meta-analysis [20–30]. All trials were published between 2004 and 2014; 9 studies were published in Chinese [22–30], and 2 were published in English [20, 21]. Two trials were multicentre trials while the others were single centre [20, 23].

**Figure 1:**
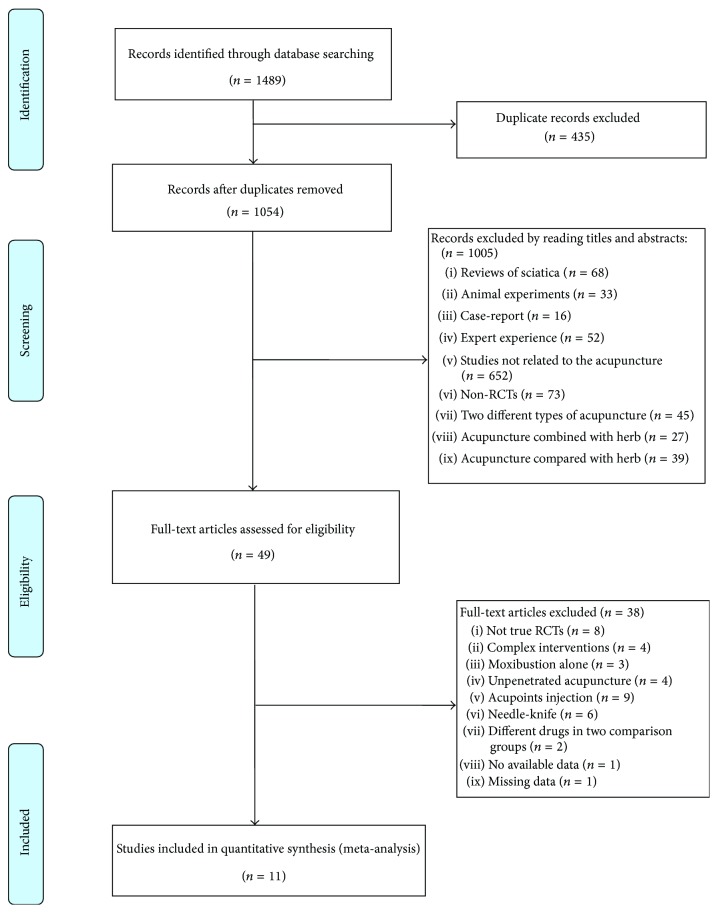
Study flow diagram.

Figure 1 uses a study flow diagram to summarize the results of the study searches.

### 3.2. Description of Studies

#### 3.2.1. Patients

We included 11 trials that enrolled a total of 962 participants in our systematic review [20–30]. Ten trials were conducted in China (932 participants) [21–30] and 1 was conducted in Pakistan (40 participants) [20]. All patients had acute or chronic sciatica; 3 trials included 180 participants diagnosed with sciatica of the nerve trunk without lumbar disc herniation and low back pain [21, 26, 30] and 8 studies (782 participants) included patients with sciatica of the nerve roots [20, 22–25, 27–29], especially caused by lumbar disc herniation. All studies stated that patients with abnormal neuralgia such as compression pain from tumour or serious infection were excluded.

The characteristics of the included studies are summarized in Table 3.

#### 3.2.2. Acupuncture Interventions

In general, all of the studies adopted a treatment theory based on traditional Chinese medicine theory and clinical experience. Many acupuncturists choose acupuncture points or corresponding acupuncture interventions based on their clinical experience during treatment. Electroacupuncture was used in most of the trials (6 studies) [20, 23–25, 28, 30], warming acupuncture was used in 3 studies [21, 27, 29], and manual needle stimulation was performed in 2 trials [22, 26]. The number of acupuncture points varied from 1 to more than 10; the most commonly used acupoints were Huantiao (GB 32), Weizhong (BL 40), and Yanglingquan (GB 34). The acupoints for each trial are shown in Table 4. The duration of interventions ranged from one to four weeks and only one trial mentioned 6 months of follow-up. The age of the patients ranged from 18 to 79 years. Eleven studies reported De-chi, a needle sensation of soreness and numbness.

#### 3.2.3. Control Interventions

In 8 trials [20–27], acupuncture was compared to conventional medications; most of the medications were nonsteroidal anti-inflammatory drugs (NSAIDs). Two studies compared acupuncture plus conventional medication to the same conventional medication alone [28, 29]. One trial used sham acupuncture in the control group [30]; the needles in this trial were inserted in nonacupuncture points (2 inches from the correct acupuncture points).

#### 3.2.4. Outcome Measure

Five studies measured pain intensity using VAS [20, 22, 25, 26, 28], which is an important assessment scale for neuralgia pain; in addition, one study used JOA [25] and one study used BRS-6 to measure pain intensity [22]. For the outcome of global assessment, 9 studies compared the patients who were cured or improved with those who were not [21–24, 26–30]. For outcome measures in most studies, “cured” means that the sciatic neuralgia resolved and the limb function recovered, while “improved” was defined as decreased sciatic neuralgia and largely normal function, and “failed” meant no symptom improvement. One study used Lasegue's sign to assess the effectiveness of the intervention [20]. The time frame of the outcome measures varied from immediately after the first treatment to 6 months after the completion of treatment.

#### 3.2.5. Risk of Bias

All of the included RCTs mentioned randomization and 7 studies reported adequate sequence generation [21–24, 26–28]; 6 trials used a table of random numbers and 1 used SPSS software to create random numbers. Three studies provided details about appropriate allocation concealment [21, 22, 26], but the related details of the remaining RCTs were unclear even after contacting the authors. Only 3 trials in the review were considered to have a low risk of bias for outcome assessors blinding [23, 26, 30]. Because of the nature of acupuncture, none of the included RCTs blinded the acupuncturists and the patients. One RCT reported 6 drop-outs but did not provide any explanation of the reasons for this [23].

### 3.3. Effects of Acupuncture

**Figure 2:**
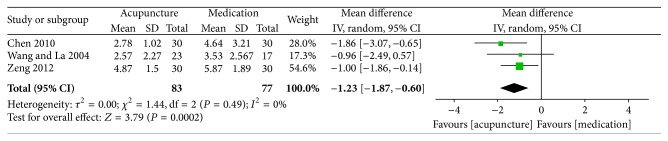


**Figure 3:**
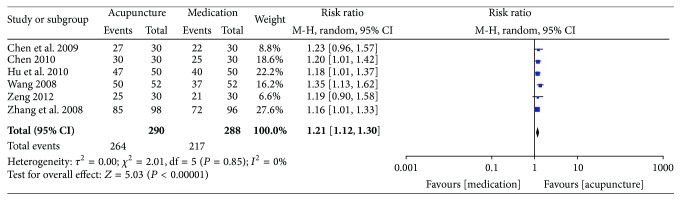


**Figure 4:**
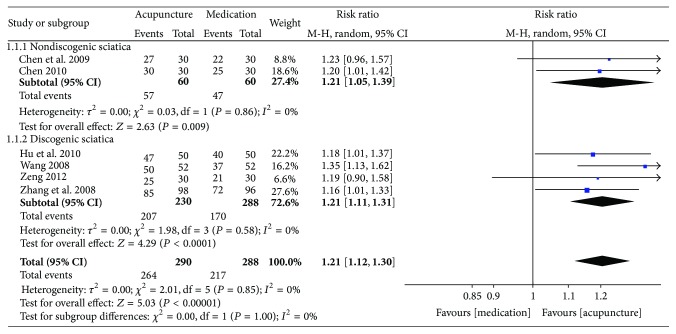


**Figure 5:**
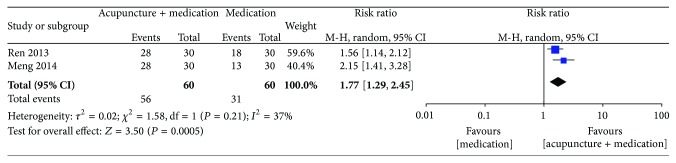


The key results from the included trials are summarized in Figures 2–5.

#### 3.3.1. Acupuncture versus Drugs 

*Visual Analogue Scale (VAS).* In terms of pain intensity related to leg/lumbago pain, 4 studies involving 222 participants contributed VAS data for meta-analysis [20, 22, 25, 26]. Meta-analysis of 4 RCTs showed considerable heterogeneity (*I* ^2^ = 66%) between the results of the included trials; we explored this heterogeneity by excluding the trial with the longest acupuncture sessions (four weeks, which was twice as long as the others). With this trial excluded, the statistical heterogeneity was reduced (*I* ^2^ = 0%). After pooling, the data showed that acupuncture might have a better effect on pain relief than conventional medication (3 trials, 160 participants, MD −1.23, 95% CI −1.87 to −0.60, and *I* ^2^ = 0%) (Figure 2).

*Assessment of the Straight Leg Raising Test*. One study used the straight leg raising test to evaluate the effect of acupuncture and medication [20]; according to the trial, after one treatment session straight leg raising improved in both groups. While the acupuncture group improved more than the medication group, the researchers concluded that electroacupuncture was more effective than NSAIDs (diclofenac) for increasing Lasegue's sign angles (the angle of Lasegue's sign, 76.70 ± 1.63 versus 70.88 ± 2.11).

*6-Point Behavioural Rating Scale (BRS-6)*. One study [22] found that the acupuncture arm might be more effective than medication in terms of the BRS-6 score (2.07 ± 1.05 versus 2.70 ± 1.34). 

*MOS Item Short Form Health Survey (MOS SF-36).* One study used the MOS SF-36 [26]. There was a statistically significant difference between acupuncture and medication in reducing the SF-36 score (57.76 ± 15.20 versus 69.07 ± 15.08).

*Japanese Orthopaedic Association (JOA) Score. *One study used the JOA score [25]. There was a statistically significant difference between acupuncture and medication in increasing the JOA score (20.16 ± 3.55 versus 17.63 ± 3.23). 

*Global Assessment. *In terms of global assessment, 6 studies involving 578 participants used global assessment as the outcome measure [21–24, 26, 27]. Data analysis showed that the patients in the acupuncture group improved more significantly after the end of the sessions than those in the medication group (6 trials, 578 participants, RR 1.21, 95% CI 1.12 to 1.30, and *I* ^2^ = 0%) (Figure 3). Although these 6 studies included sciatica of the nerve trunk and sciatica of the nerve roots and although the meta-analysis showed no heterogeneity, we still feel that the results may have been influenced by different types of sciatica. Thus, to evaluate the efficacy of acupuncture for different types of sciatica, a subgroup analysis was conducted according to our predesigned protocol; pooling the data of these studies showed that, for sciatica of the nerve roots [22–24, 27], the therapeutic effect of acupuncture was significantly better than drugs (4 trials, 474 participants, RR 1.08, 95% CI 1.02 to 1.14, and *I* ^2^ = 0%), and for sciatica of the nerve trunk [21, 26], acupuncture can provide symptom relief (2 trials, 104 participants, RR 1.21, 95% CI 1.05 to 1.39, and *I* ^2^ = 0%) (Figure 4).

#### 3.3.2. Acupuncture versus Sham Acupuncture

*Global Assessment. *One study [30] reported that acupuncture provided more improvement in global assessment than sham acupuncture (29/30 versus 22/30).

#### 3.3.3. Acupuncture Plus Drugs versus the Same Drugs

*Pain Intensity. *One study [28] involving 60 participants reported that acupuncture plus medication was significantly more effective than medication alone in providing pain relief (pain intensity on VAS; 3.04 ± 0.53 versus 4.82 ± 0.62) after two acupuncture treatment sessions.

*Global Assessment. *Two studies [28, 29] reported that acupuncture plus conventional medication provided significantly more improvement than conventional medication alone (2 studies, 87 participants, RR 1.77, 95% CI 1.29 to 2.45, and *I*² = 37%) (Figure 5). 

*Adverse Effects. *Three of 11 trials reported adverse effects [23, 24, 26]. In one trial, patients in the acupuncture group reported 3 cases of hypodermal bleeding while 21 patients in the medication group reported gastrointestinal problems including nausea, stomach ache, dyspepsia, and headache [23]. In another trial, no adverse events were reported in the acupuncture group while 5 patients in the medication group reported gastrointestinal problems [24]. One trial reported 2 adverse events in the acupuncture group and no adverse events in the control group [26]. Although acupuncture appears to be associated with few adverse effects, the evidence is limited.

## 4. Discussion

### 4.1. Summary of Main Results

Sciatica affects many people and is a common reason for seeking medical advice. It has considerable economic consequences in terms of health care resources and lost productivity [6, 37]. In this systematic review, although we made an extensive literature search, because of language barriers and the predefined inclusion criteria, only 11 studies of acupuncture for sciatica were eligible for our systematic review and meta-analysis. After combining 3 RCTs [20, 22, 26], the results of the meta-analysis showed that acupuncture may be more effective than NSAIDs (ibuprofen, meloxicam, and diclofenac) in decreasing the VAS for leg pain/lumbago, (3 trials, 160 participants, MD −1.23, 95% CI −1.87 to −0.60, and *I* ^2^ = 0%) and 1 RCT concluded that acupuncture plus an NSAID (ibuprofen) was superior to the same NSAIDs alone (pain intensity on VAS; 3.04 ± 0.53 versus 4.82 ± 0.62) [28]. Although one prior systematic review reported that no evidence exists for NSAIDs being superior to placebo [38], NSAIDs were still suggested for pain control by the clinical guideline for diagnosis and treatment of sciatica from the Dutch College of General Practice [39]. In addition, VAS pain intensity score is the primary outcome of interest in sciatica; the score in the acupuncture group was significantly lower than that in the NSAIDs group (pooled MD = 1.23), but, considering that the sample sizes of the included trials were small, it is difficult to draw conclusions. Moreover, there was sparse information in these RCTs regarding the processes of randomization and allocation concealment, and only 3 of the RCTs blinded the statisticians [23, 26, 30], which may have led to a considerable risk of bias. Therefore, the present findings suggest that acupuncture may be more effective than NSAIDs in relief of leg pain/lumbago, but the evidence is limited. In addition, compared with medication, acupuncture appears to be more effective regarding physical signs, motor function, or quality of life measured by other scales such as the JOA, BRS-6, SF-36, and Lasegue's sign. However, because these 4 trials reported the outcomes separately [20, 22, 25, 26] and meta-analysis was not possible for one trial, and taking into account the small sample sizes of the included trials, it was difficult to make robust conclusions.

In terms of global assessment, the combined results of 6 RCTs showed that acupuncture was superior to medication in improving global assessment (6 trials, 578 participants, RR 1.21, 95% CI 1.12 to 1.30, and *I* ^2^ = 0%) [21–24, 26, 27], and acupuncture plus medication was better than the same medication alone (2 studies, 87 participants, RR 1.77, 95% CI 1.29 to 2.45, and *I* ^2^ = 37%) in improving the global assessment [28, 29]. It is important to explain that we chose global assessment as the primary outcome of interest in our published protocol [18]; however, most of the included RCTs used “Criteria of diagnosis and therapeutic effect of diseases and syndromes in traditional Chinese medicine” to report outcomes on the basis of an ordinal assessment (“cured,” “improved,” and “failed”). This makes it difficult to evaluate and save global assessment as the primary outcome; hence we redesigned global assessment to be one of the secondary outcomes. Compared with sham acupuncture (2 inches from the real acupuncture point), 1 RCT suggested that real acupuncture may be more effective in global assessment (29/30 versus 22/30) [30]. Meta-analysis was impossible for a single trial with a small sample size; therefore, it is difficult to draw a conclusion without powerful evidence. However, the results may suggest that the treatment of acupuncture points may be relatively specific for sciatica.

Acupuncture appears to be associated with fewer adverse effects compared with NSAIDs. Six of the included 11 RCTs mentioned adverse events and only 2 of them reported adverse events in the acupuncture group (5 cases of hypodermal bleeding) [23, 26]. Therefore acupuncture is safe for treating patients with sciatica. Even though acupuncture is associated with adverse effects such as hypodermal bleeding, in contrast to the gastrointestinal adverse effects associated with NSAIDs, acupuncture might be an option method for patients who cannot tolerate the adverse effects to the digestive system. More information is needed to better evaluate the adverse effects of the two interventions.

Given the characteristics of sciatica, the presence of inflammation and well-established nociceptive pathways may necessitate a threshold dose or duration of acupuncture treatment prior to clinical effect [38, 40]. This is supported by pathophysiologic and anatomic studies illustrating how the sustained nociceptive input caused by sciatica can have profound effects on the central nervous system, causing pathologic neuroplastic changes. The controlled stimulation of peripheral nociceptors with acupuncture may reverse such pathologic neuroplasticity in the central nervous system, especially when administered over a prolonged period [40].

The quality of trials is not sufficiently high and efforts to improve trial reporting are necessary; subsequent trials should comply with the CONSORT statement and STRICTA recommendations [32, 40]. Outcome measures should not be confined to global assessment. VAS, NRS, quality of life and mobility function, and follow-up should also be addressed in the future trials. As a prior published systematic review related to acupuncture reported that the cost effectiveness is another insufficiently researched aspect of acupuncture RCTs [41], the above issues should be taken into consideration to allow clinicians and patients to make evidence-based treatment decisions.

### 4.2. Applicability of Evidence

In this systematic review, 2 of the included trials were multicentre in nature [20, 23]; the other trials were of small sample size and most of the trials had poor methodological quality, lacking details regarding blinding and allocation concealment. The majority of the included trials used global assessment to measure outcomes and interventions varied greatly in terms of the acupuncture intervention methods, treatment periods, and the location of acupuncture points; the statistical results may have varied. In addition to the different acupuncture intervention methods, we also must take variations in the area of medications into consideration. Although most of the conventional medication was NSAIDs, variations in the effects of NSAIDs cannot be ignored.

### 4.3. Limitations of This Review

This review may be limited by the inherent methodological limitations of the included RCTs.

We chose to consider acupuncture treatment regardless of the frequency of administration, duration of each session, and number and location of acupoints in our published protocol. Any of these variables may have influenced the effects of acupuncture.

Because of the language barrier, we were unable to include other trials that may have met our inclusion criteria.

## 5. Conclusion

In conclusion, the results of this systematic review suggest that the use of acupuncture may more effectively relieve leg pain/lumbago and improve global assessment of sciatica when compared with NSAID (ibuprofen, meloxicam, and diclofenac) treatment. Moreover, adjuvant acupuncture may enhance the effect of medications in leg pain/lumbago relief. To patients, acupuncture points appear more effective than nonacupoints. Acupuncture is relatively safe and is rarely associated with serious adverse events in patients with sciatica. However, this meta-analysis was lacking in relevant and rigorous RCTs. Because the evidence was limited, higher quality and more rigorously designed clinical trials with larger sample sizes will be needed to further confirm our findings.

## Supplementary Material

PRISMA stands for Preferred Reporting Items for Systematic Reviews and Meta-Analyses. It is an evidence-based minimum set of items for reporting in systematic reviews and meta-analyses, which consists of a 27-itm checklist and a four-phase flow diagram, and the PRISMA can be used as a basis for reporting or appraising systematic reviews. Here, this systematic review and meta-analysis is reported in accordance with the PRISMA statement.

## Figures and Tables

**Table 1:** Search strategy used in PubMed database.

Number	Search items
1	randomized controlled trial.pt
2	controlled clinical trial.pt
3	randomized.ti,ab
4	randomly.ti,ab
5	groups.ti,ab
6	trial.ti,ab
7	or 1–6
8	acupuncture.ti,ab
9	electro-acupuncture.ti,ab
10	elongated needle.ti,ab
11	three edged needle.ti,ab
12	(fire needle or warming needle).ti,ab
13	auricular acupuncture.ti,ab
14	abdominal acupuncture.ti,ab
15	warm acupuncture.ti,ab
16	pyonex.ti,ab
17	or 8–16
18	sciatica.ti,ab
19	sciatic neuralgia.ti,ab
20	ischialgia.ti,ab
21	ischioneuralgia.ti,ab
22	discogenic sciatica.ti,ab
23	bilateral sciatica.ti,ab
24	disc herniation-induced sciatica.ti,ab
25	or 18–24
26	7 and 17 and 25

This search strategy will be modified as required for other electronic databases.

**Table 2:** Acupuncture interventions in the included studies based on the STRICTA recommendation.

Author	Insertion depth	Response sought	Details of needling Stimulation method	Retention time	Needle type	Treatment regimen	Practitioner background
Wang and La 2004 [20]	NR	De-chi response manual	EA	25 min	NR	1 session once a day for 7 days	Physician
Chen et al. 2009 [21]	NR	De-chi response manual	WA	20–35 min	0.3 × 60 mm	3 sessions once daily for 10 days	NR
Zeng 2012 [22]	60 mm (GB 30/BL 54) others 25 mm	De-chi response manual	Manipulated every 10 min, pricking blood	30 min	0.3 × 75 mm	2 sessions once daily for 10 days	NR
Zhang et al. 2008 [23]	40–60 mm	De-chi response manual	Manipulated every 10 min and EA	20 min	0.3 × 40–75 mm	2 sessions once daily for 10 days	Professional acupuncturists
Hu et al. 2010 [24]	60 mm (GB 30) others 40 mm	De-chi response manual	Manipulated every 10 min and EA	30 min	0.3 × 50–75 mm	2 sessions once daily for 10 days	NR
Du et al. 2009 [25]	45–60 mm	De-chi response manual	EA	45 min	0.45 × 75 mm	4 sessions 3 times per week	NR
Chen 2010 [26]	40–75 mm	De-chi response manual	Manipulated every 10 min	30 min	0.3 × 25–40 mm	2 sessions 3 times per week	NR
Wang 2008 [27]	NR	De-chi response manual	WA	NR	0.4 × 75 mm	2 sessions once daily for 10 days	NR
Meng 2014 [28]	NR	NR	EA	30 min	NR	2 sessions once daily for 7 days	Qualified acupuncturist
Ren 2013 [29]	40–75 mm	De-chi response manual	WA	30 min	NR	1 session once a day for 10 days	NR
Zhao 2004 [30]	50–75 mm	De-chi response manual	EA	30 min	0.25 × 75 mm	2 sessions once a day for 10 days	NR

NR: not reported, De-chi: a needle sensation of soreness and numbness, EA: electroacupuncture, WA: warm acupuncture, and STRICTA: standards for reporting interventions in controlled trials of acupuncture.

**Table 3:** Summary of studies included in the review.

Author	Design	Number of subjects	Intervention type (A)	Control group (B)	Treatment regimen	Follow-up periods	Outcome measure	Results reported	Adverse events
Wang and La 2004 [20]	Parallel, 2 arms	40 (23/17)	EA	Diclofenac sodium 50 mg tid for 7 d	1 session once a day for 7 days	NR	(1) Laseque's sign angles (2) VAS	(1) (A) versus (B): 76.67 ± 1.63 versus 70.88 ± 2.11 (2) (A) versus (B): 25.71 ± 2.27 versus 35.33 ± 2.57	NR

Chen et al. 2009 [21]	Parallel, 3 arms	90 (30/30/30)	WA	(1) Nimesulide 0.1 g bid (2) Acupuncture points injection	3 sessions once daily for 10 days	NR	(1) Response rate (2) PT	(1) (A) significantly better than (B) (*P* < 0.05, 90% versus 73.33%) (2) 2.62 ± 0.59 versus 1.54 ± 0.39	NR

Zeng 2012 [22]	Parallel, 2 arms	60 (30/30)	MA Pricking blood	Ibuprofen 3 mg bid + Vb_2_ 10 mg tid	2 sessions once daily for 10 days	NR	(1) Response rate (2) BRS-6 (3) VAS	(A) significantly better than (B) (1) (*P* < 0.05, 83.3% versus 70%) (2) 4.87 ± 1.50 versus 5.87 ± 1.89 (3) 2.07 ± 1.05 versus 2.70 ± 1.34	NO

Zhang et al. 2008 [23]	Parallel, 2 arms	194 (98/96)	EA	Meloxicam 7.5 mg qd	2 sessions once daily for 10 days	NR	Response rate	(A) significantly better than (B) (*P* < 0.05, 86.53% versus 75%)	3 patients reported hypodermal bleeding in intervention group; 21 patients in control group reported GI problems

Hu et al. 2010 [24]	Parallel, 2 arms	100 (50/50)	EA	Meloxicam 7.5 mg qd	2 sessions once daily for 10 days	Six month	Response rate	(A) significantly better than (B) (*P* < 0.05, 94% versus 80%); after half year follow-up 83% versus 70%	5 patients in control group reported GI problems

Du et al. 2009 [25]	Parallel, 2 arms	62 (32/30)	EA	Diclofenac sodium 75 mg qd	4 sessions 3 times per week	NR	(1) JOA for total score (2) VAS	(1) 20.16 ± 3.54 versus 17.63 ± 3.23 (2) 2.12 ± 1.12 versus 2.10 ± 1.39	NR

Chen 2010 [26]	Parallel, 2 arms	60 (30/30)	MA	Ibuprofen 0.2 g tid + prednisone 30 mg qd	2 sessions 3 times per week	NR	(1) Response rate (2) VAS (3) MOS SF-36	(1) (A) significantly better than (B) (*P* < 0.05, 100% versus 83.3%) (2) VAS 2.78 ± 1.02 versus 4.64 ± 3.21 (3) MOS SF-36 for GH 57.76 ± 15.20 versus 59.07 ± 15.08	2 patients reported hypodermal bleeding in intervention group

Wang 2008 [27]	Parallel, 2 arms	104 (52/52)	WA	Ibuprofen 0.6 g bid + Vb_1_ 30 mg tid	2 sessions once daily for 10 days	NR	Response rate	(A) significantly better than (B) (*P* < 0.05, 96.2% versus 71.2%)	NR

Meng 2014 [28]	Parallel, 2 arms	60 (30/30)	EA + drugs (same as control group)	Ibuprofen 20 mg bid + Vb_1_ 30 mg tid	2 sessions once daily for 7 days	NR	(1) Response rate (2) VAS	(1) (A) significantly better than (B) (*P* < 0.05, 93.33% versus 43.33%) (2) 3.04 ± 0.53 versus 4.28 ± 0.62	NO

Ren 2013 [29]	Parallel, 2 arms	60 (30/30)	WA + drugs (same as control group)	Mannitol 150 mL + dexamethasone 10 mg i.v.gtt and mecobalamin tablets 0.5 mg I.M.	1 session once a day for 10 days	NR	Response rate	(A) significantly better than (B) (*P* < 0.05, 93.3% versus 60%)	NR

Zhao 2004 [30]	Parallel, 2 arms	60 (30/30)	EA	Sham acupuncture	2 sessions once a day for 10 days	NR	Response rate	(A) significantly better than (B) (*P* < 0.05, 97.7% versus 73.3%)	NO

NR: not reported, EA: electroacupuncture, WA: warm acupuncture, i.v.gtt: intravenous drip, I.M.: intramuscular injection, VAS: Visual Analogue Scale, SF MPQ: Short-Form McGill Pain Questionnaire, PT: pain threshold, JOA: Japanese Orthopaedic Association score, BRS-6: 6-point behavior rating scale, MOS SF-36: the medical outcome study item short form health survey, GI: gastrointestinal, and GH: general health.

**Table 4:** Acupoints of each trial.

Wang and La 2004 [20]	Huantiao (GB 30), Weizhong (BL 40)

Chen et al. 2009 [21]	Shenshu (BL 23), Dachangshu (BL 25), Huantiao (GB 30), Weizhong (BL 40), and Kunlun (BL 60)

Zeng 2012 [22]	Huantiao (GB 30), Zhibian (BL 54), Chengfu (BL 36), Fengshi (GB 31), Weizhong (BL 40), Yanglingquan (BL 67), Chengshan (BL 57), Xuanzhong (GB 39), Kunlun (BL 60), and Zulinqi (GB 41)

Zhang et al. 2008 [23]	Jiaji (EX-B2), Yaoyangguan (DU 3), Huantiao (GB 30), and Yanglingquan (BL 67)

Hu et al. 2010 [24]	Yaoyangguan (DU 3), Shiqizhui (EX-B7), Huantiao (GB 30), Yanglingquan (BL 67), Weizhong (BL 40), and Chengshan (BL 57)

Du et al. 2009 [25]	Jiaji (EX-B2)

Chen 2010 [26]	Jiaji (EX-B2), Zhibian (BL 54), Huantiao (GB 30), Yinmen (BL 37), Weizhong (BL 40), Chengshan (BL 57), and Kunlun (BL 60)

Wang 2008 [27]	Jiaji (EX-B2), Zhibian (BL 54), Weizhong (BL 40), and Yanglingquan (BL 67)

Meng 2014 [28]	Jiaji (EX-B2), Huantiao (GB 30), Juegu (GB 39), Weizhong (BL 40), and Zhibian (BL 54)

Ren 2013 [29]	Dachangshu (BL 25), Shenshu (BL 23), Mingmen (DU 4), Guanyuanshu (BL 26), Qihaishu (BL 24), Zhibian (BL 54), Huantiao (GB 30), and Jiaji (EX-B2)

Zhao 2004 [30]	Huantiao (GB 30), Weizhong (BL 40)
